# Multiple Targets of 3-Dehydroxyceanothetric Acid 2-Methyl Ester to Protect Against Cisplatin-Induced Cytotoxicity in Kidney Epithelial LLC-PK1 Cells

**DOI:** 10.3390/molecules24050878

**Published:** 2019-03-01

**Authors:** Dahae Lee, Ki Hyun Kim, Won Yung Lee, Chang-Eop Kim, Sang Hyun Sung, Kyo Bin Kang, Ki Sung Kang

**Affiliations:** 1 School of Pharmacy, Sungkyunkwan University, Suwon 16419, Korea; pjsldh@naver.com (D.L.); khkim83@skku.edu (K.H.K.); 2 College of Korean Medicine, Gachon University, Seongnam 13120, Korea; wonyung21@naver.com (W.Y.L.); eopchang@gachon.ac.kr (C.-E.K.); 3 College of Pharmacy and Research Institute of Pharmaceutical Sciences, Seoul National University, Seoul 08826, Korea; shsung@snu.ac.kr; 4 College of Pharmacy, Sookmyung Women’s University, Seoul 04310, Korea

**Keywords:** cisplatin, MAPKs, nephrotoxicity, apoptosis

## Abstract

Chronic exposure to cisplatin, a potent anticancer drug, causes irreversible kidney damage. In this study, we investigated the protective effect and mechanism of nine lupane- and ceanothane-type triterpenoids isolated from jujube (*Ziziphus jujuba* Mill., Rhamnaceae) on cisplatin-induced damage to kidney epithelial LLC-PK1 cells via mitogen-activated protein kinase (MAPK) and apoptosis pathways. Cisplatin-induced LLC-PK1 cell death was most significantly reduced following treatment with 3-dehydroxyceanothetric acid 2-methyl ester (3DC2ME). Additionally, apoptotic cell death was significantly reduced. Expression of c-Jun N-terminal kinase (JNK), extracellular signal-regulated kinase (ERK), and p38 was markedly suppressed by 3DC2ME, indicating inhibition of the MAPK pathway. Treatment with 3DC2ME also significantly reduced expression of active caspase-8 and -3, Bcl-2-associated X protein (Bax), and B cell lymphoma 2 (Bcl-2), indicating the inhibition of apoptosis pathways in the kidneys. We also applied the network pharmacological analysis and identified multiple targets of 3DC2ME related to MAPK signaling pathway and apoptosis.

## 1. Introduction

The kidney is essential for health and quality of life in humans as it maintains homeostasis of extracellular electrolytes, fluid balance, and blood pressure [1,2]. *cis*-Diamminedichloroplatinum II (CDDP or cisplatin), is a potent anticancer drug used in the treatment of a variety of cancers such as solid or hematologic tumors [3,4]. However, the application of cisplatin is limited by its potential to damage renal tubular epithelial cells, and thus cause acute kidney injury [5,6].

Studies have reported that cisplatin-induced damage to renal tubular epithelial cells is mediated by activating molecular mechanisms of DNA damage [7,8,9], oxidative [10,11,12] and nitrosative stress [11,13], inflammation [14,15], mitogen-activated protein kinase (MAPK) [14], and apoptosis [8,14]. Natural products and their compounds may reduce this damage via antioxidant, anti-apoptotic, and anti-inflammatory properties in a wide variety of cells [5,16,17,18].

In this regard, we have evaluated the protective effects of a medicinal plant and its compounds against cisplatin-induced kidney cell toxicity to investigate the use of natural products that are effective but less toxic. Several studies have shown that kidney damage is reduced by treatment with triterpenoids such as betulinic acid [19,20], total triterpenoids from *Psidium guajava* leaves [21], oleanolic acid [22,23,24], and the synthetic triterpenoids RTA 405 [25] and RTA 408 [26]. Results indicate that the use of triterpenoids is an effective approach to reducing kidney injury. Jujube (*Ziziphus jujuba* Mill., Rhamnaceae) has been used as a traditional herbal medicine and food in Asia for thousands of years [27,28,29]. Various biological activities have been reported for jujube and its extracts, including anticancer, anti-oxidative, anti-inflammatory, hepatoprotective, gastrointestinal protective, neuroprotective, and anti-obesity effects. A number of phytochemicals have been isolated from *Z. jujuba* including polyphenols, triterpenoids, and polysaccharides, and these metabolites are reported to contribute to the bioactivity of jujube [27,28,30].

Triterpenoids are known as major constituents of *Z. jujuba*. Most triterpenoids isolated from *Z. jujuba* are pentacyclic triterpenoids, especially of the ursane, oleanane, lupane, and ceanothane type. Our previous studies revealed that lupane-type triterpenoids from *Cornus walteri* and lanostane-type triterpenoids from *Poria cocos* exhibit nephroprotective effects on cisplatin-induced proximal tubular damage [31,32] Thus, we hypothesized that lupane- and ceanothane-type triterpenoids from *Z. jujuba* would also display nephroprotective effects against cisplatin-induced damage in kidney epithelial LLC-PK1 cells and investigated this further. Moreover, we explored the mechanism of action of the triterpenoid at systems level by predicting potential targets and applying network pharmacological analysis.

## 2. Results

### 2.1. Protective Effects of Nine Triterpenoids from Z. jujuba Against Cisplatin-Induced LLC-PK1 Cell Death in LLC-PK1 Cells

**Figure 1:**
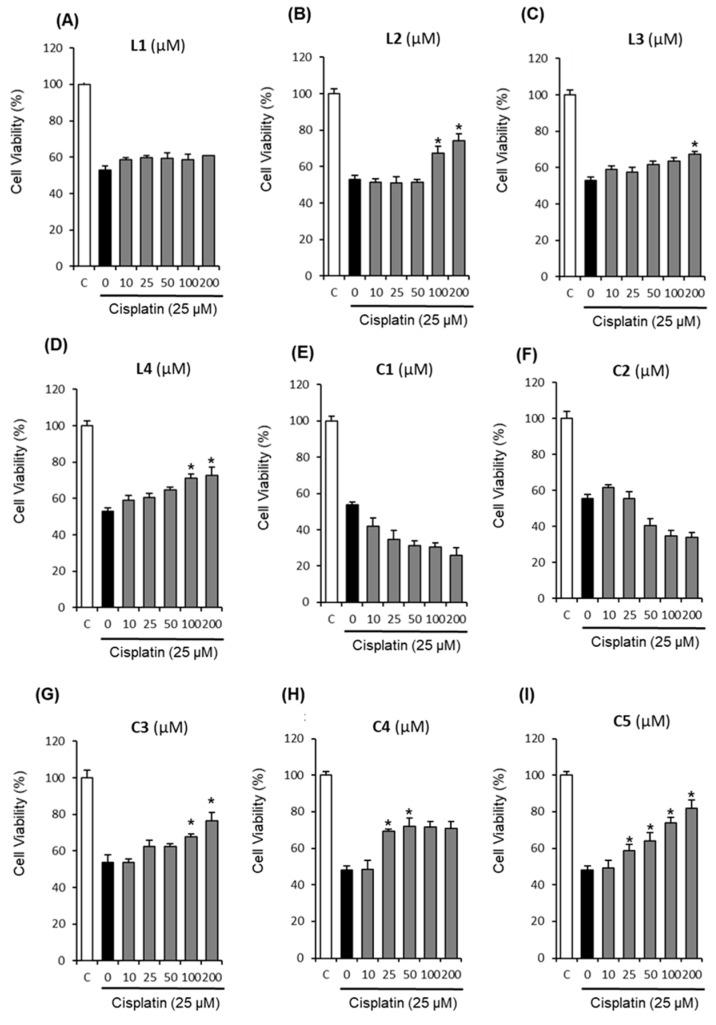
Comparison of the effects of nine triterpenoids on viability LLC-PK1 cells exposed to 25 μM cisplatin for 24 h by MTT assay. (**A**) Effect of L1 against cisplatin-induced LLC-PK1 cell Death in LLC-PK1 cells. (**B**) Effect of L2 against cisplatin-induced LLC-PK1 cell Death in LLC-PK1 cells. (**C**) Effect of L3 against cisplatin-induced LLC-PK1 cell Death in LLC-PK1 cells. (**D**) Effect of L4 against cisplatin-induced LLC-PK1 cell Death in LLC-PK1 cells. (**E**) Effect of C1 against cisplatin-induced LLC-PK1 cell Death in LLC-PK1 cells. (**F**) Effect of C2 against cisplatin-induced LLC-PK1 cell Death in LLC-PK1 cells. (**G**) Effect of C3 against cisplatin-induced LLC-PK1 cell Death in LLC-PK1 cells. (**H**) Effect of C4 against cisplatin-induced LLC-PK1 cell Death in LLC-PK1 cells. (**I**) Effect of C5 against cisplatin-induced LLC-PK1 cell Death in LLC-PK1 cells. Control cells were treated with the vehicle only (mean ±SD, * *p* < 0.05 compared to the control).

To evaluate the protective effects of nine triterpenoids isolated from the roots of *Z. jujuba*, LLC-PK1 cells were treated with cisplatin after pre-treatment with indicated concentrations of triterpenoids for 2 h. Based on the results of our previous study [33], cisplatin was applied at 25 μM that inhibited LLC-PK1 cell viability by 40%. Cell viability was then measured via Ez-Cytox cell viability assay. As shown in Figure 1, cell viability decreased to 47.98 ± 2.48% following treatment with 25 μM cisplatin for 24 h when compared with the control cells. By contrast, almost all of the compounds (except L1, C1, and C2) displayed protective effects in a dose-dependent manner, while L1 induced no changes in LLC-PK1 cell viability (Figure 1A). Furthermore, C1 and C2 induced toxicity in LLC-PK1 cells and decreased cell viability in a dose-dependent manner (Figure 1E,F). Interestingly, of the nine triterpenoids tested, 3DC2ME (C5) showed the strongest protective effect (Figure 1I). The viability of LLC-PK1 cells was significantly increased by pre-treatment with 3DC2ME in a concentration-dependent manner. The maximum protective effect was observed at a concentration of 200 μM (81.82 ± 4.60% cell viability, Figure 1I). Mechanistic studies were then carried out with 3DC2ME, as treatment with this compound appeared to be sufficiently protective from cell death.

### 2.2. Protective Effects of 3DC2ME Against Cisplatin-Induced Apoptosis in LLC-PK1 Cells

**Figure 2:**
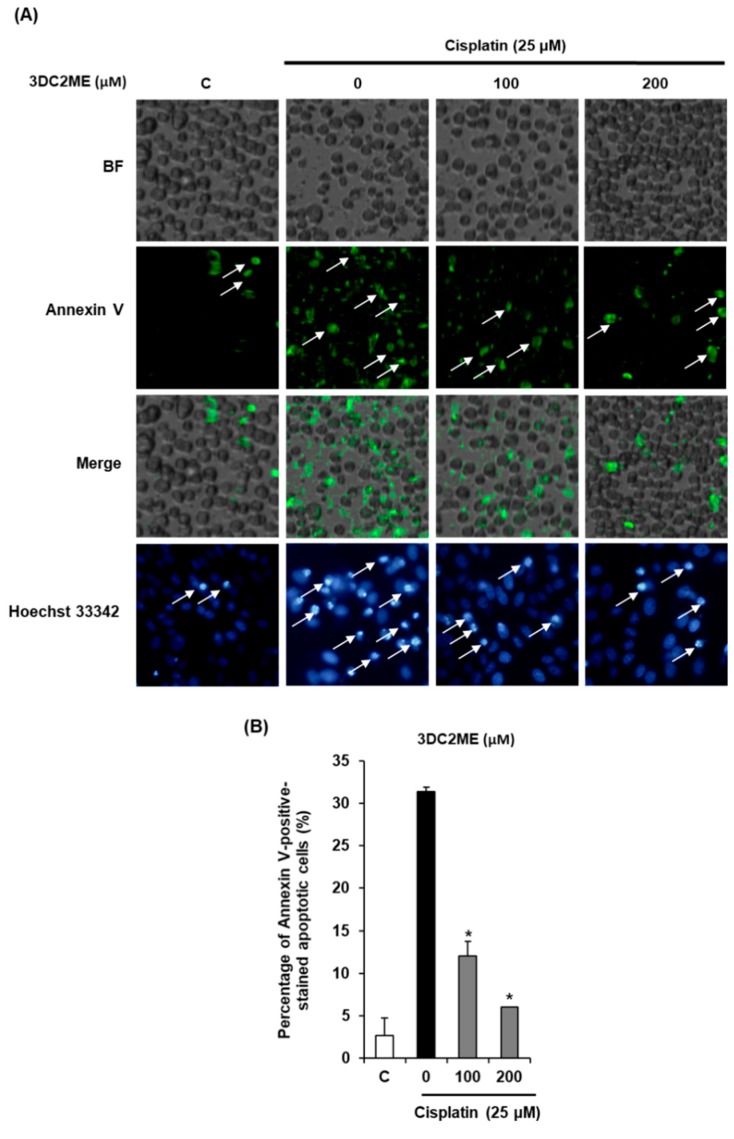
Effects of 3DC2ME on apoptosis in LLC-PK1 cells exposed to 25 μM cisplatin for 24 h (image-based cytometric assay and Hoechst 33342 staining). (**A**) Representative images for apoptosis, (**B**) percentage annexin V-positive-stained apoptotic cells. Control cells were treated with the vehicle only (mean ±SD, * *p* < 0.05 compared to the control).

We then explored whether 3DC2ME could decrease cisplatin-induced apoptosis in LLC-PK1 cells. Cells were exposed to 25 μM cisplatin in the presence or absence of 3DC2ME and stained with annexin V conjugated with Alexa Fluor 488, and Hoechst 33342. As shown in Figure 2, the percentage of annexin V-positive cells indicating apoptosis was significantly increased to 31.33 ± 0.57% by treatment with 25 μM cisplatin, whereas it was decreased by treatment with 100 μM and 200 μM 3DC2ME to 12.00 ± 1.73% and 6.00 ± 0.00%, respectively (Figure 2A,B). In addition, after cisplatin treatment, apoptotic morphological changes in the cells were observed by fluorescence microscopy after staining with Hoechst 33342, a stain used to observe DNA condensation during apoptosis, whereas such changes were reduced by treatment with 100 μM and 200 μM 3DC2ME (Figure 2A).

### 2.3. Protective Effects of 3DC2ME on Expression of MAPK and Apoptosis Proteins in Cisplatin-Induced Damage in LLC-PK1 Cells

**Figure 3:**
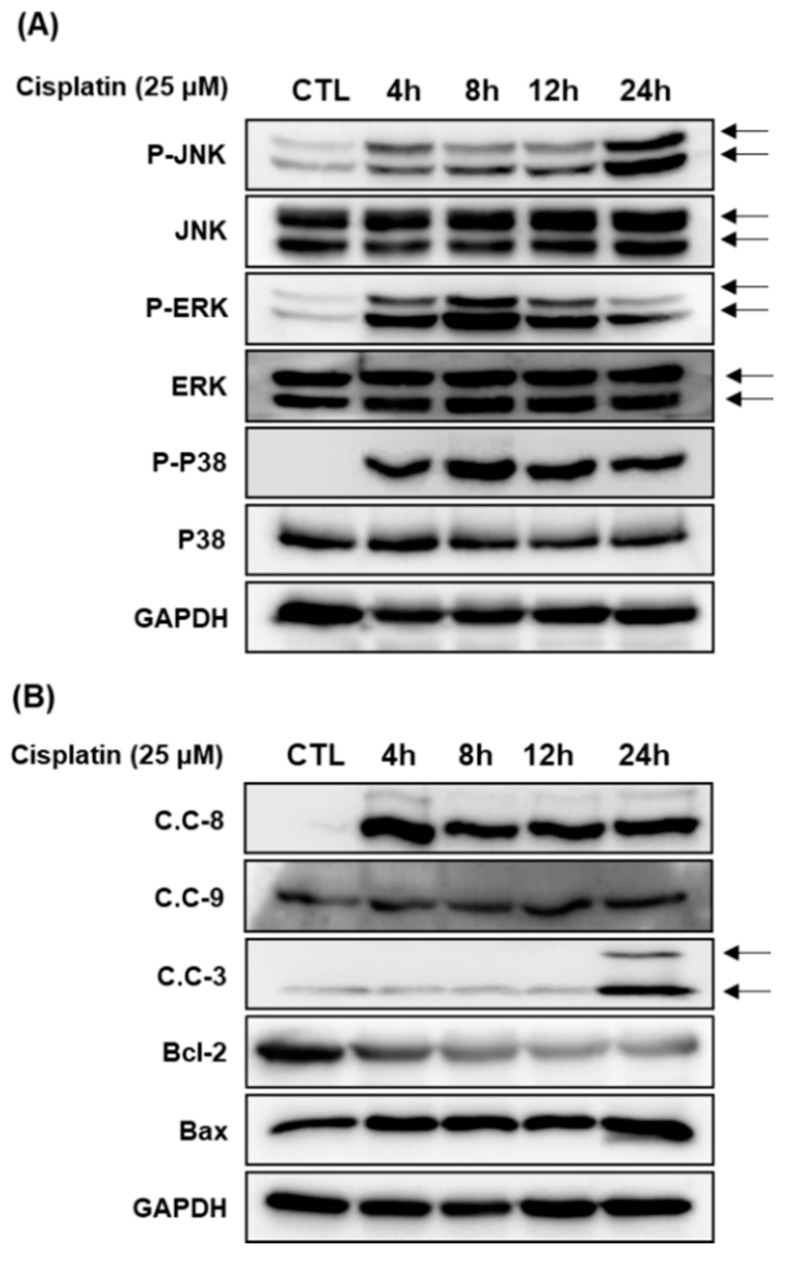
Time-course (4 h, 8 h, 12 h, and 24 h) protein expression of proteins associated with (**A**) MAPK and (**B**) apoptosis pathways in LLC-PK1 cells exposed to 25 μM cisplatin by western blot. Control cells were treated with the vehicle only (mean ±SD, * *p* < 0.05 compared to the control). CTL, cisplatin; phosphor-c-Jun N-terminal kinase, P-JNK; phosphor-extracellular signal-regulated kinase, p-ERK; glyceraldehyde 3-phosphate dehydrogenase, GAPDH; cleaved caspase-8, C.C-8; cleaved caspase-9, C.C-9; cleaved caspase-3, C.C-3.

To elucidate the molecular mechanism of the protective effects of 3DC2ME, LLC-PK1 cells were exposed to 25 μM cisplatin for 24 h followed by western blot analysis to evaluate expression of MAPK signaling proteins (c-Jun N-terminal kinase (JNK), extracellular signal regulated kinase (ERK), and p38) and apoptosis pathway proteins (caspase-3, -8, -9, Bcl-2-associated X protein (Bax), and B cell lymphoma 2 (Bcl-2)) at various time-points (4 h, 8 h, 12 h, and 24 h). We examined their activation profiles over later time points. Our studies revealed that LLC-PK1 cells exposed to 25 μM cisplatin displayed increased phosphorylation of JNK, EKR, and p38 at 4 h (Figure 3A). Cleavage of caspase-8 and -9 and activation of Bax were increased at 4 h post treatment. Activation of Bcl-2 decreased at 4 h and cleavage of caspase-3 increased at 24 h (Figure 3B).

**Figure 4:**
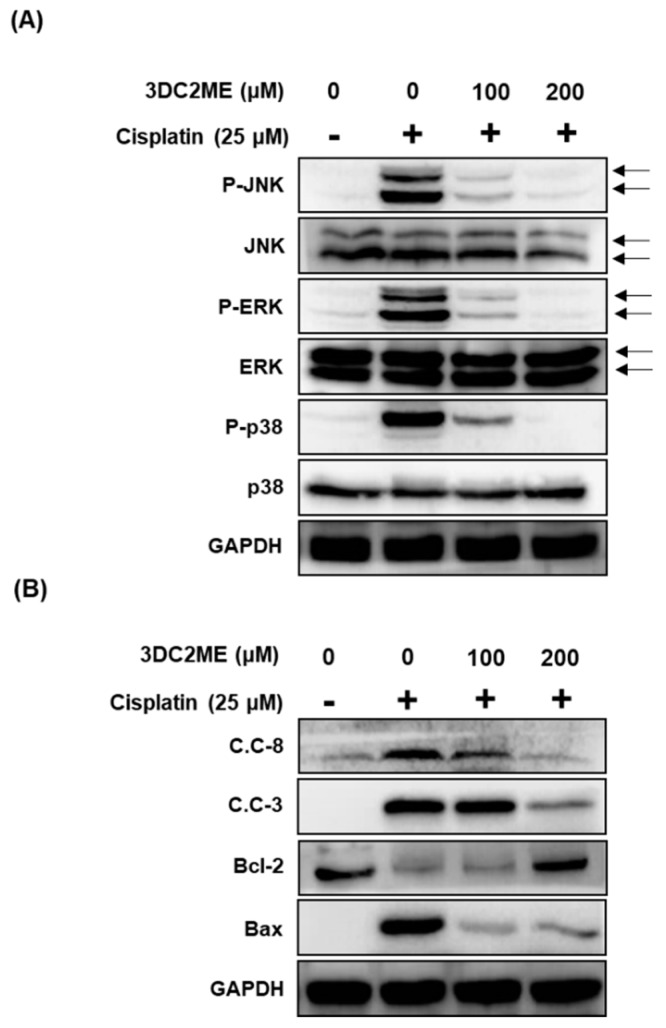
Effects of 3DC2ME on protein expression of proteins associated with (**A**) MAPK and (**B**) apoptosis pathways in LLC-PK1 cells exposed to 25 μM cisplatin by western blot. Control cells were treated with the vehicle only (mean ±SD, * *p* < 0.05 compared to the control). 3-Dehydroxyceanothetric acid 2-methyl ester, 3DC2ME; phosphor-c-Jun N-terminal kinase, P-JNK; phosphor-extracellular signal-regulated kinase, p-ERK; glyceraldehyde 3-phosphate dehydrogenase, GAPDH; cleaved caspase-8, C.C-8; cleaved caspase-9, C.C-9; cleaved caspase-3, C.C-3.

We then evaluated the effects of 3DC2ME on expression of MAPK and apoptosis proteins in cisplatin-induced damage in LLC-PK1 cells. LLC-PK1 cells were exposed to 25 μM cisplatin for 24 h with or without 100 μM and 200 μM 3DC2ME followed by western blot. Co-treatment with 100 μM and 200 μM 3DC2ME was shown to completely inhibit the activation and expression of MAPK (Figure 4A) and apoptosis proteins (Figure 4B).

### 2.4. Effects of Combined Treatment with Inhibitors of MAPK Pathways (SB203580 and U0126) and 3DC2ME on Cisplatin-Induced LLC-PK1 Cell Death

**Figure 5:**
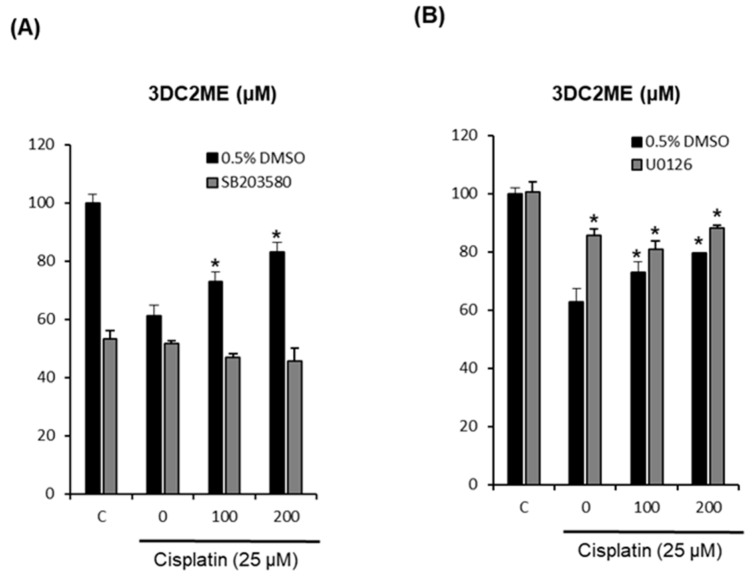
Effects of (**A**) SB203580 and (**B**) U0126 on the protective effects of 3DC2ME in cisplatin-induced cytotoxicity in LLC-PK1 cells. An MTT assay was performed on LLC-PK1 cells exposed to 25 μM cisplatin for 24 h. Control cells were treated with the vehicle only (mean ±SD, * *p* < 0.05 compared to the control).

To elucidate the involvement of MAPK pathways, we investigate the effects of combined treatment with 3DC2ME and inhibitors of MAPK pathways on cisplatin-induced LLC-PK1 cell death. LLC-PK1 cells were treated with 25 μM cisplatin and/or SB203580 (p38 inhibitor) or U0126 (ERK inhibitor) after pre-treatment with 100 μM and 200 μM 3DC2ME for 24 h. Cell viability was then measured via Ez-Cytox cell viability assay. As shown in Figure 5A, SB203580 induced no changes in LLC-PK1 cell viability while the protective effects of 3DC2ME correlated with earlier results (Figure 1I). Interestingly, as shown in Figure 5B, treatment with U0126 attenuated cisplatin-induced LLC-PK1 cell death and this protective effect was increased by the treatment of 100 μM and 200 μM 3DC2ME. The treatment of 25 μM cisplatin decreased cell viability to 62.72 ± 4.57%, but U0126 showed a protective effect, significantly increasing the cell viability to 85.69 ± 2.19% (Figure 5B). Moreover, combined treatment with U0126 and 3DC2ME showed protective effects, but no significant synergistic protective effects. The viability of LLC-PK1 cells was significantly increased to 88.12 ± 0.97% after combined treatment with U0126 and 200 μM 3DC2ME (Figure 5B).

### 2.5. Effect of Co-Treatment with 3DC2ME and Cisplatin in HeLa Human Cervical Carcinoma Cells

**Figure 6:**
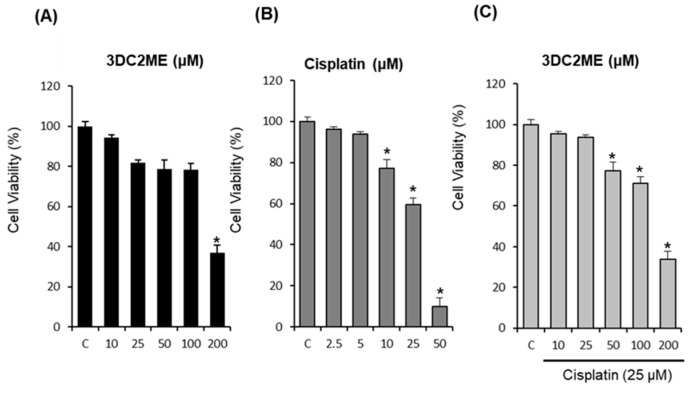
Effects of co-treatment with 3DC2ME and cisplatin in HeLa human cervical carcinoma cells for 24 h (MTT assay). (**A**) Effect of 3DC2ME on HeLa cell viability. (**B**) Effect of cisplatin on HeLa cell viability. (**C**) Effect of co-treatment with 3DC2ME and cisplatin on HeLa cell viability. Control cells were treated with the vehicle only (mean ±SD, * *p* < 0.05 compared to the control).

To evaluate the cytotoxic effects of co-treatment with 3DC2ME and cisplatin, HeLa cells were treated with indicated concentrations of 3DC2ME and cisplatin for 24 h. Cell viability was then measured via Ez-Cytox cell viability assay. Cell viability decreased to 36.77 ± 4.03% after treatment with 200 μM 3DC2ME for 24 h compared with control cells (Figure 6A). In addition, cisplatin induced cytotoxicity at concentrations of 25 μM (59.49 ± 1.97%) and 50 μM (10.04 ± 0.48%, Figure 6B). However, combined treatment with 200 μM 3DC2ME and 25 μM cisplatin showed no significant synergistic cytotoxic effects (Figure 6C).

### 2.6. Network Pharmacological Approach

Network pharmacological analyses were conducted to elucidate the system-level mechanism of 3DC2ME. We obtained 274 potential targets of 3DC2ME using the machine learning model (see methods). Six target genes of 12 tested biomarker-related genes were found in the predicted target lists (Bcl-2, caspase-3, caspase-8, MAPK1, MAPK10, and MAPK14). The accordance rate was significantly higher than the chance level (*p*-value = 4.00 × 10^−5^, hypergeometric test), supporting the reliability of our in silico model.

**Figure 7:**
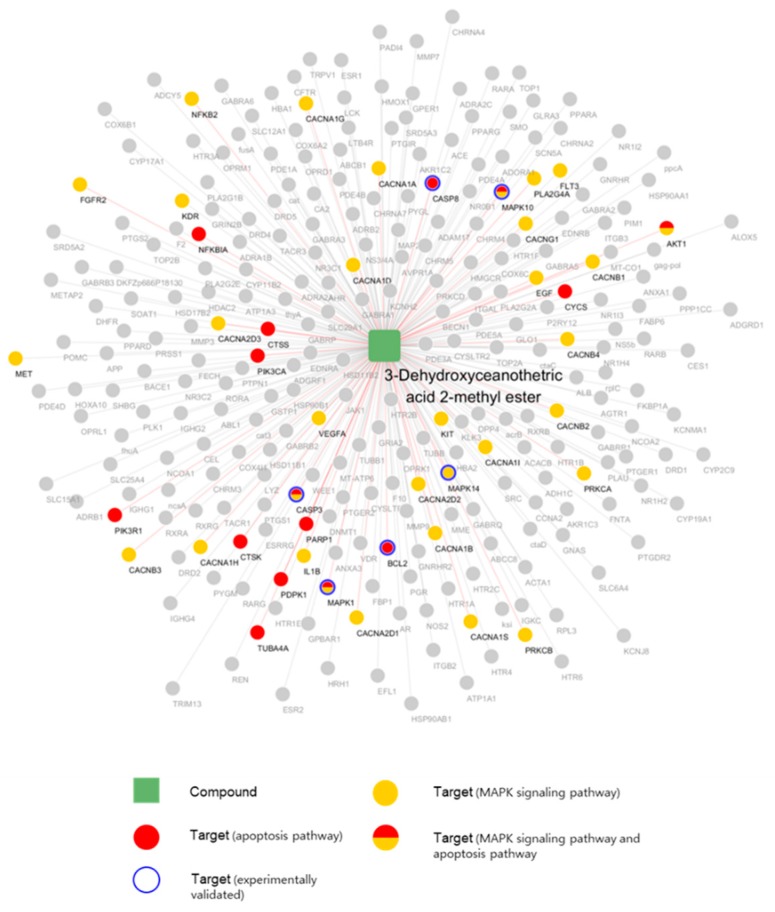
Compound-target network of 3DC2ME. Rectangles represent compounds, and circles represent the targets. Nodes related to MAPK signaling or apoptosis in the KEGG pathway are colored.

**Figure 8:**
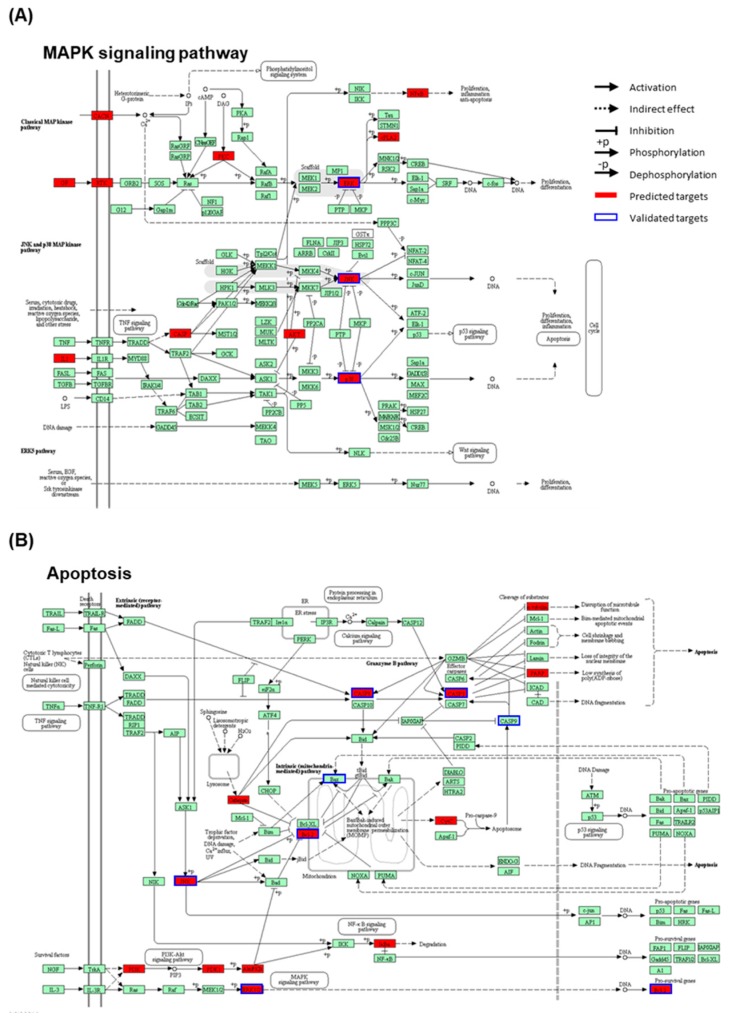
Representation of targets of 3DC2ME in (**A**) MAPK and (**B**) apoptosis pathways. The pathway maps were constructed using KEGG mapper.

We constructed and visualized the compound-target network of 3DC2ME using Cytoscape [34]. The nodes and edges were colored to indicate the related node with the pathways of interest (Figure 7). The numbers of related targets in MAPK signaling pathway and apoptosis pathway were 27 and 15, respectively. Six targets, caspase-3, MAPK1, MAPK10, MAPK14, Bcl-2, and caspase-8, were related to both pathways. Pathway enrichment analysis revealed 274 potential targets were significantly associated with MAPK and apoptosis pathway (adjusted *p*-value = 1.78 × 10^−16^, and 9.08 × 10^−10^, respectively). We also mapped the targets of 3DC2ME in MAPK and apoptosis pathway using KEGG (Figure 8) [35]. Multiple targets related to MAPK and apoptosis pathway supports the system-level mechanism of 3DC2ME.

## 3. Discussion

Earlier studies reported that cisplatin-induced renal cell death involved MAPK [14,36,37,38] and apoptosis [37,38,39,40,41,42,43] signaling pathways. Our previous studies also showed that cisplatin-induced apoptosis was mediated through MAPK and apoptosis signaling pathways, and that this cytotoxicity was ameliorated by treatment with triterpenoids from natural products in LLC-PK1 cells [37,38]. The nephroprotective effects of triterpenoids isolated from *Z. jujuba* against anticancer drug-induced damage in kidney cells have not been reported. Hence, we report our investigation of the nephroprotective effects of triterpenoids isolated from *Z. jujuba* for the first time in the present study. We hypothesized that 3DC2ME isolated from *Z. jujuba* could attenuate cisplatin-induced proximal tubular damage through inhibition of MAPK and apoptosis pathways. We therefore investigated the role of MAPK and apoptosis signaling in renoprotection associated with 3DC2ME isolated from *Z. jujuba* using an in vitro model. The protective effects of 3DC2ME from *Z. jujuba* against cisplatin-induced LLC-PK1 cell death are evidenced by alleviated cell death. In addition, morphological changes related to protective effects were also investigated via annexin V staining and Hoechst 33342 staining. Annexin V detects phosphatidylserine expression in cells undergoing apoptosis [44,45]. Hoechst 33342 detects nuclear shrinkage, chromatin condensation, and nuclear fragmentation [32]. All of these characteristics are indicator of apoptosis. Apoptosis has most frequently been referred to in cisplatin-induced renal cell death. In this study, apoptosis was observed when cells were exposed to cisplatin, which induces LLC-PK1 cell death, whereas this was reduced by treatment with 3DC2ME. Thus, it is important to study the mechanism of protection of 3DC2ME.

The MAPK signaling pathway is considered a critical regulator of signal transduction that serves to regulate diverse cellular responses to extracellular stimuli [46,47,48]. Cisplatin can activate MAPK signaling pathways, including JNK, ERK, and p38 in proliferative or toxic renal injury. JNK and p38 contribute to renal epithelial cell death [46,47,48]. On the contrary, ERK contributes to renal epithelial cell growth and differentiation. However, several recent studies demonstrate that ERK also contributes to renal epithelial cell death [14,36,46,47,49]. Inhibition of ERK with inhibitors such as U0126 or PD98059 attenuates cisplatin-induced LLC-PK1 cell death [14,36,43]. Our studies revealed that cisplatin induced activation of MAPK. On the contrary, 3DC2ME was shown to completely inhibit the activation of MAPK. Furthermore, treatment with U0126 attenuates cisplatin-induced LLC-PK1 cell death and this protective effect was better than that of treatment with 100 μM and 200 μM 3DC2ME.

Bcl-2 family proteins are considered critical regulators of apoptosis [40,50,51]. Bcl-2 is an anti-apoptotic Bcl-2 family protein that suppresses apoptotic cell death and promotes cell survival by preserving the integrity of the mitochondrial membrane [40,50,51]. Bax is known as a pro-apoptotic Bcl-2 family protein that promotes apoptotic cell death by increasing permeability of the mitochondrial membrane [40,41,51]. Our studies revealed that cisplatin induced apoptosis via downregulation of Bcl-2 and up-regulation of Bax. On the contrary, 3DC2ME was shown to completely inhibit cisplatin-induced apoptosis via up-regulation of Bcl-2 and downregulation of Bax.

Caspases are known to act downstream of Bax/Bcl-2 and play a critical role in apoptosis [51,52,53]. The cell death pathway is divided into two well-characterized arms. One is a receptor-mediated pathway that is initiated by activation of cell death receptors. It activates caspase-8, which proteolytically activates caspase-3. The other is a mitochondrial-dependent pathway that is initiated by cytochrome c release from the mitochondria. It activates caspase-9, which also proteolytically activates caspase-3 [42,54]. Our studies also revealed that cisplatin induced apoptosis via activation of executioner caspase-3 and initiator caspase-8 and -9. On the contrary, 3DC2ME was shown to completely inhibit cisplatin-induced apoptosis via inhibition of caspase-3 and -8 cleavage.

An additional study confirmed that co-treatment with 3DC2ME and cisplatin does not decrease the anti-proliferative activity of cisplatin on HeLa human cervical carcinoma cells. 3DC2ME may therefore prevent cisplatin-induced cytotoxicity without affecting its anticancer properties.

We applied a novel network pharmacological analysis in this study. The results not only support our results in vitro, but also gives opportunities to understand the system-level mechanisms of 3DC2ME. Our results suggest that the nephroprotective effects of 3DC2ME are likely achieved by modulation of multiple targets of multiple pathways.

## 4. Materials and Methods

### 4.1. Preparation of Triterpenoids from Z. jujuba

**Figure 9:**
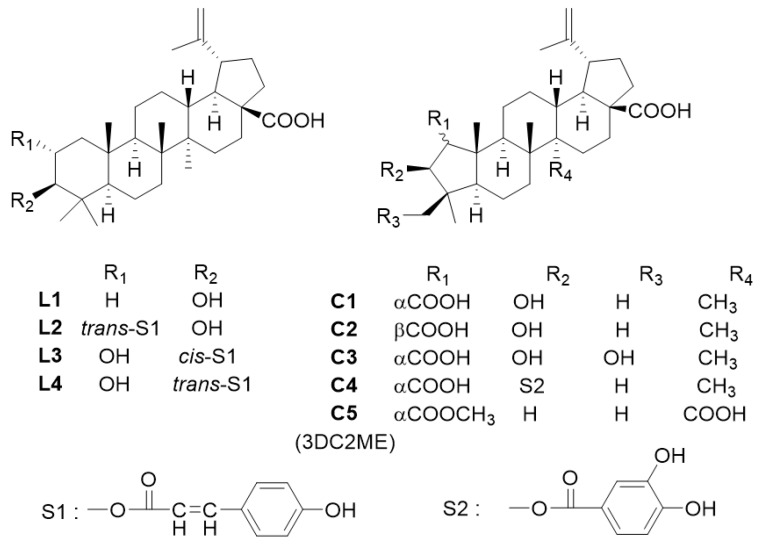
The chemical structures of four lupane-type (L1–L4) and five ceanothane-type (C1–C5) triterpenoids isolated from *Z. jujuba*.

Four lupane- (L1, betulinic acid; L2, 2-*O*-trans-*p*-coumaroyl alphitolic acid; L3, 3-*O*-*cis*-*p*-coumaroyl alphitolic acid; L4, 3-*O*-*trans*-*p*-coumaroyl alphitolic acid) and five ceanothane-type triterpenoids (C1, ceanothic acid; C2, epiceanothic acid; C3, 24-hydroxyceanothic acid; C4, 3-*O*-protocatechuoyl ceanothic acid; C5, 3-dehydroxyceanothetric acid 2-methyl ester (3DC2ME)) were prepared in our previous study (Figure 9) [55]. Briefly, these were isolated through a series of column chromatographic techniques from the methanol extract of *Z. jujuba* roots. Chemical structures of isolated compounds were identified via nuclear magnetic resonance (NMR) spectroscopy (AVANCE 600 spectrometers, Bruker, Billerica, MA, USA), and the purities of compounds were determined to be greater than 95% via NMR and liquid chromatography–mass spectrometry (LC–MS; Waters Xevo G2 QTOF mass spectrometer, Waters MS Technologies, Manchester, UK) analyses.

### 4.2. Cell Culture

LLC-PK1 pig kidney epithelial cells and HeLa human cervical carcinoma cells were purchased from the American Type Culture Collection (ATCC, Manassas, VA, USA). Both cell types were cultured in Dulbecco’s modified Eagle medium (ATCC) supplemented with 10% fetal bovine serum (Invitrogen, Grand Island, NY, USA), 1% penicillin/streptomycin, and 4 mM l-glutamine in a humidified incubator with 5% CO_2_ at 37 °C.

### 4.3. Measurement of Cell Viability

Cells were seeded at a density of 1 × 10^4^ cells on 96-well plates and incubated for 24 h. The compounds of 100 mM were used as stock solutions in dimethyl sulfoxide (Sigma-Aldrich, St. Louis, MO, USA), and these were further diluted with medium to the desired concentration. The cells were pre-treated with indicated concentrations of compounds for 24 h. Untreated cells were used as controls. After incubation, the Ez-Cytox cell viability assay kit (Daeil Lab service, Seoul, Korea) was used to assess cell viability [56]. Briefly, after treatment, cells were incubated with 2 μL Ez-Cytox reagent for 30 min at 37 °C. Cell viability was then assessed by measuring absorbance at 450 nm using a microplate reader (PowerWave XS; Bio-Tek Instruments, Winooski, VT, USA).

### 4.4. Image-based Cytometric Assay

An image-based cytometric assay was used to assess annexin V-positive-stained apoptotic cells [57]. Briefly, after treatment, harvested cells were resuspended in binding buffer (Life Technologies, Carlsbad, CA, USA). A 100 μL aliquot was incubated with 5 μL annexin V Alexa Fluor 488 (Invitrogen, Temecula, CA, USA) for 30 min in the dark. Stained cells were counted via a Tali image-based cytometer (Invitrogen) using annexin V Alexa Fluor 488 staining (Life Technologies).

### 4.5. Cell Staining with Hoechst 33342

Staining with Hoechst 33342, a DNA-binding dye, was used to assess DNA condensation during apoptosis, a process that does not occur during necrosis [58]. Briefly, after treatment, cells were incubated with 2 μL Hoechst 33342 solution for 10 min at 37 °C. Stained cells were then analyzed via fluorescence microscopy.

### 4.6. Western Blotting Analysis

Western blotting analysis was used to assess expression of target proteins. Briefly, after treatment, the harvested cells were washed twice with cold phosphate-buffered saline, and total cell lysates were prepared with radio immunoprecipitation assay buffer (RIPA buffer, Cell Signaling Technology, Inc., Beverly, MA, USA) supplemented with 1 × EDTA-free protease inhibitor cocktail and 1 mM phenylmethylsulfonyl fluoride (PMSF) according to the manufacturer’s instructions.

Protein content was quantified via bicinchoninic acid (BCA) protein assay, and 20 μg, along with molecular weight markers, was separated via 10% sodium dodecyl sulfate polyacrylamide gel electrophoresis (SDS–PAGE), for 90 min at 110 V. This was then transferred to a polyvinylidene fluoride (PVDF) transfer membrane and immunoblotted with corresponding antibodies. Immunodetection was performed using the ECL Advance Western Blotting Detection Reagents (GE Healthcare, Cambridge, UK) and a FUSION Solo Chemiluminescence System (PEQLAB Biotechnologie GmbH, Erlangen, Germany) [59].

### 4.7. Network Pharmacological Analysis

In silico prediction of comprehensive target profiles is the first step using network pharmacology approaches. We predicted the potential targets based on the machine learning models proposed by Yu et al., which incorporates the chemical, genomic and pharmacological information to derive the classifier [31]. Previously known drug-target interactions were obtained to train the models from the latest version of Drugbank (5.1.0, accessed on Mat 1st 2018, Edmonton, Canada) [60]. DRAGON 7.0 (Pisa, Italy) and PROFEAT 2016 (Singapore, Singapore) were used to calculate chemical, protein descriptors, respectively [61]. Ensemble method (extremely randomized trees) was applied to predict novel drug-target interactions.

To decipher the system-level mechanism of 3DC2ME, we conducted network pharmacological analysis. Compound-target network was constructed by linking 3DC2ME and all predicted targets using the drug-target interactions information. Pathway enrichment analysis was conducted using Kyoto Encyclopedia of Genes and Genomes database (KEGG, http://www.genome.jp/kegg/) and Enrichr API [62].

### 4.8. Statistical Analysis

The data are presented as means ± standard deviations (SD). Statistical significance was determined using analysis of variance (followed by Bonferroni multiple testing correction), and hypergeometric test. *P*-values smaller than 0.05 were considered statistically significant.

## 5. Conclusions

These findings, taken together, indicate that the protective effects of 3DC2ME from *Z. jujuba* against cisplatin-induced renal epithelial cell death are mediated by regulation of MAPK and apoptosis pathways.
